# The Influence of Infection and Colonization on Outcomes in Inpatients With COVID-19: Are We Forgetting Something?

**DOI:** 10.3389/fpubh.2021.747791

**Published:** 2021-11-10

**Authors:** Jose Luis Alfonso-Sanchez, Adriana Agurto-Ramirez, María A. Chong-Valbuena, Isabel De-Jesús-María, Paula Julián-Paches, Luis López-Cerrillo, Hilary Piedrahita-Valdés, Martina Giménez-Azagra, José María Martín-Moreno

**Affiliations:** ^1^Preventive Medicine Service, Hospital General Universitario de Valencia, Valencia, Spain; ^2^Department of Preventive Medicine and Public Health, School of Medicine, University of Valencia, Valencia, Spain; ^3^Biomedical Research Institute INCLIVA, Hospital Clinico Universitario de Valencia, Valencia, Spain

**Keywords:** risk factors, SARS-CoV-2, COVID-19, disease management, COVID epidemiology, germ colonization

## Abstract

The COVID-19 epidemic has been a great challenge to health systems and especially hospitals. A prospective observational epidemiological study was planned as of February 26, 2020 in a tertiary hospital in the Valencia region. The total number of patients followed up with complete information during the first year was 2,448. Among other variables, the comorbidities of the patients were collected (and grouped in the Charson index), the stay in the intensive care unit (ICU), the co-infections, and the colonizations. Data on nosocomial infections due to said virus were also collected. The median days from the onset of symptoms to diagnosis were 4 + 4.6, while an additional 4.4 days had to pass for the patients to be admitted to the ICU. The factors associated with a higher risk of death were those with coinfection, especially with *Candida auris* [odds ratio (OR): 4.6], a situation that also occurred in the ICU (OR: 3.18). Charlson Index comorbidity and *C. auris* colonization were also very important both in general hospitalization and in the ICU.

## Introduction

Since the first known cases were described in Wuhan at the end of 2019 (1), the infectious disease caused by SARS-CoV-2 and christened as COVID-19 by the World Health Organization (2) has become an unprecedented pandemic. It has tested the limits of scientific and health system capacity with regard to understanding SARS-CoV-2 transmission mechanisms, physiopathology, the body's humoral and cellular immune response, and clinical presentation; building diagnostic capacity; determining the best pharmacological and non-pharmacological treatments; and developing truly effective and comprehensive control strategies. Fortunately, new knowledge is gradually being generating in response to the unrelenting need imposed by the circumstances.

COVID-19 encompasses a wide clinical spectrum, as it can present as anything from an asymptomatic or mild infection to severe pneumonia and respiratory failure, with the potential to develop into a multiorgan syndrome. Its complications can even lead to death, with rapid onset of acute respiratory distress syndrome in some patients (3).

Prior to the pandemic, antimicrobial resistance in bacteria and fungi was among the main health threats globally (4–6). The impact of antibacterial and antimycotic resistance can be minimized through adherence to recommendations on the administration of antimicrobials and the implementation of infection control measures. These practices are based on the active detection of colonization with resistant pathogens as well as compliance with strict precautions regarding the isolation of carriers to prevent horizontal transmission among patients (7, 8). Optimizing prescriptions of antimicrobials can be challenging in patients with COVID-19, as the clinical severity, imaging characteristics, and laboratory parameters complicate the differentiation between bacterial coinfection and the effects of SARS-CoV-2 itself. It is important to characterize the underlying risk present in the patient's situation; tools like the Charlson comorbidity index can be useful in defining diverse clinical conditions predictive of mortality (9) and that can influence prognosis and confer added risk.

Finally, as part of the treatment strategy and considering the above, it is necessary to adapt infection control programs and administration of antimicrobial agents in real time to evolving and updated scientific evidence (7). The aim of this study is to determine the importance that infection and/or colonization with multidrug-resistant germs has on outcomes in COVID-19, taking into account patient comorbidities.

## Materials and Methods

### Data and Analysis

The study was carried out in a tertiary hospital in Valencia, Spain. This Hospital has a Preventive Medicine Service that is dedicated to the control of hospital isolations and infections. It also has an accredited Microbiology Service in its procedures and cultures.

This was an observational epidemiological study with prospective follow-up; it was still ongoing at the time of writing. Patients were enrolled upon admission to hospital for COVID-19, starting on 27 February 2020; we report on outcomes to 26 February 2021, that is, at one natural year from study commencement.

Data were collected on a daily basis from our own electronic healthcare databases, with continuous cross-referencing, for all COVID-19 patients admitted to a public tertiary hospital in the province of Valencia (Spain) (*N* = 2,448). The hospital is the reference center for microbiological testing for a large part of the province of Valencia (more than 500,000 inhabitants), especially Health Department number 9.

The study included all patients admitted for more than 24 h; identifying information was anonymized in accordance with regulations on patient confidentiality and personal data protection laws.

Variables analyzed were: age, sex, mean length of hospital stay for COVID-19 (days), mean length of stay in patients admitted to the intensive care unit (ICU), date of symptoms onset, date of discharge for COVID-19, type of coinfection, and/or colonization, classified as: *Candida auris*, multidrug-resistant germs, or both; Charlson index (scoring according to that index), time from symptoms onset to hospital admission (days), and type of discharge (death, yes/no). In this study co-infection is when two or more germs are in or on the body and make you sick, which results in signs and symptoms such as fever, pus from a wound, a high white blood cell count, diarrhea, or pneumonia. Colonization means germs are on the body but do not make you sick. People who are colonized will have no signs or symptoms (10).

The Charlson index included 19 comorbidities for which points were assigned to patients, in line with the classification (9). The group of multidrug-resistant germs comprised all the germs classified as such in the hospital antibiogram.

Some variables were recoded: age was transformed into a categorical variable by 10-year age groups in the descriptive analysis and length of hospital stay (ward/ICU) was categorized in intervals of 10 days. Charlson index scores were considered both as an ordinal variable (1, 2, 3,.6), with all scores of 7 or more grouped into a single category, and as a dichotomous variable (0 points vs. ≥1 point).

The length of hospital stay was considered only for the time admitted to the corresponding unit, until the patient was considered “COVID-19-free” or was discharged to their residence, nursing home, or even another hospital ward when an additional pathology required it.

### Statistical Analysis

Quantitative variables are described as means (standard deviation, SD), but for the distribution from symptoms onset to hospital admission including nosocomial transmission we used median and SD. The normality of the distribution was tested using the Kolmogorov-Smirnov statistic, and means were compared first with Levene's test of equal variances and then with the student's *t*-test. Variables were grouped, covariances studied, and simple and multiple logistic regressions performed, with analysis of possible interactions and confounding. For small, non-parametric samples, we used the Mann-Whitney U- and Wilcoxon W-tests.

Independence and multicollinearity of the variables were assessed using the Durbin Watson and variance inflation factor tests. Goodness-of-fit was also assess using the Hosmer-Lemeshow test. We calculated odds ratios (ORs) with their 95% confidence intervals (CIs) for all variables, including dummy variables. *P*-values of < 0.05 were considered statistically significant.

Analyses were undertaken with SPSS software for Windows (version 26.0, IBM Corp., Armonk, NY).

### Study Setting and Population

A specific COVID-19 unit with established treatment protocols was created to attend patients.

To assess antibiotic resistance, gram-positive germs were tested with betalactams, aminoglucosides, and other antibiotics like vancomycin, levofloxacin, and linezolid. Gram-negative germs were exposed to betalactams, piperacillin/tazobactam, imipenem, aminoglucosides, and others such as ciprofloxacin and fosfomycin. Resistance to meropenem (minimum inhibitory concentration ≥8 mg/L) was considered indicative of resistance to all carbapenems (11).

For *C. auris*, susceptibility was interpreted according to the cutoffs proposed by the US Centers for Disease Control and Prevention (12). All strains of *C. auris* considered in the present study demonstrated resistance to amphotericin B and had shown greater susceptibility to azoles and echinocandins. In addition to collecting test swabs, microbiological samples were taken in case of clinical suspicion of infection. The microorganisms cultured from the blood samples, nasopharyngeal specimens, or urine were identified by means of MALDI-TOF mass spectrometry, antimicrobial susceptibility tests were performed with the Vitek2 system (Biomerieux, Craponne, France), and antifungal testing employed the microdilution method of the Clinical and Laboratory Standards Institute and the Sensititre YeastOne panel (Thermo Scientific, Waltham, MA, USA).

Bacterial infection was defined as an acute infection that included both the coinfection at the time of presentation and the secondary infection that emerged over the course of the illness and the hospital stay, as we could not always distinguish between these. We considered colonization based on the same criteria.

Microbiological eradication was defined by negative cultures during follow-up plus improvement in clinical and laboratory parameters. Clinical cure was defined as clinical improvement without evidence of microbiological eradication.

The primary outcome was colonization or the development of infection with multidrug-resistant germs, *C. auris*, or both in inpatients with COVID-19.

This study protocol was conducted according to the guidelines of the Declaration of Helsinki and approved by the Valencia University General Hospital Ethics Committee. The information obtained was treated with absolute confidentiality, respecting the principles of the Declaration of Helsinki. Participants' data were anonymized upon extraction. All patients, when invited to be included in the health system through their personalized identification number, gave their authorization to the Regional Ministry of Health for their information to be used for research purposes, in compliance with data protection regulations.

## Results

A total of 2,448 patients were admitted and discharged over the study period, including 364 people admitted to the ICU. Table 1 presents patients' baseline characteristics. Approximately 55% were men, and the mean age was 66.7 years (SD 18.3). On average, women were older than men (68.9 vs. 64.8 years, *p* > 0.05). The age group with the most representation was 71–80 years, comprising 21% of the sample. Admission trends showed clear waves of COVID-19 infections. The first, starting in March 2020, showed the largest month-to-month increase in admissions in the entire study period. Admissions fell to very low levels until August 2020, when cases again started to increase at rates of 1,000%. Admissions increased at low rates, especially at the beginning of December 2020, until a third wave of infections started, resulting in an increased rate of admissions (85%) in January 2021, before starting to fall again in February 2021. Mean length of hospital stay in the total sample was 11.6 days (SD 11.5), while for ICU patients it was 13.9 days (SD 14.5). Notably, 36.9% of the patients had a Charlson comorbidity score of 0. It should be underlined that in patients with comorbidity, COPD, and diabetes were the most frequent pathologies, together accounting for slightly more than 50%. On the other hand, 15% of the patients admitted for COVID-19 had a co-infection with MR germs, the most frequent germs being *Escherichia coli* and *Klebsiella pneumoniae*, which together accounted for 50% of all co-infections. In addition to the above, it is worth noting that the overall mortality rate for all inpatients, including ICU patients, was 19.4%.

**Table 1:** Baseline characteristics of inpatients with COVID-19, February 2020 to February 2021.

		***N***	**%**	**Cumulative %**
**Gender**	Men	1,345	54.9	54.9
	Women	1,103	45.1	100
**Age group (years)**	0–10	15	0.6	0.6
	11–20	8	0.3	0.9
	21–30	51	2.1	3.0
	31–40	145	5.9	8.9
	41–50	277	11.3	20.3
	51–60	384	15.7	35.9
	61–70	407	16.6	52.6
	71–80	514	21.0	73.6
	81–90	480	19.6	93.2
	91–100	164	6.7	99.9
	>100	3	0.1	100
**Month of admission**	Feb-20	3	0.1	0.1
	Mar-20	227	9.3	9.4
	Apr-20	119	4.9	14.3
	May-20	17	0.7	15.0
	Jun-20	8	0.3	15.3
	Jul-20	7	0.3	15.6
	Aug-20	105	4.3	19.9
	Sep-20	87	3.6	23.4
	Oct-20	168	6.9	30.3
	Nov-20	327	13.4	43.6
	Dec-20	391	16.0	59.6
	Jan-21	727	29.7	89.3
	Feb-21	262	10.7	100
**Length of hospital stay (including ICU stay), in days**	0–10	1,542	63.0	63.0
	11–20	622	25.4	88.4
	21–30	176	7.2	95.6
	31–40	42	1.7	97.3
	41–50	31	1.3	98.6
	51–60	16	0.7	99.2
	61–70	5	0.2	99.4
	71–80	7	0.3	99.7
	81–90	1	0	99.8
	91–100	1	0	99.8
	>100	5	0.2	100
**Charlson comorbidity**	No	903	36.9	36.9
	Yes	1,545	100	
	Chronic pulmonary disease	479	31	31
	Diabetes	309	20	51
	Moderate or severe liver diseases	185	11,97	62,97
	Diabetes with chronic complications	171	11,07	74,04
	Rheumatologic disease	139	9	83,04
	Other comorbidities	262	16,96	100
**Coinfection with MDR**	Non	2,079	84.93	84.93
	Yes	369	15.07	100,00
**Type of MDR**	*Escherichia coli*	101	27.37	27.37
	*Klebsiella pneumoniae*	85	23.04	50.41
	*Pseudomona aeruginosa*	65	17.62	68.03
	*Staphilococus aereus*	50	13.54	81.57
	*Acinetobacter baumannii/Haemolyticus*	41	11.11	92.68
	Others	27	7.32	100,00
**Type of discharge**	No death	1,974	80.6	
	Death	474	100	

*MDR, multidrug resistance*.

Table 2 presents data on the interval from symptoms onset to hospital admission, as reported by patients, as well as the date of admission. Although the data set was widely dispersed, the three groups with the shortest interval (0, 1, and 2 days from onset to admission) made up over 36% of the total. There was also a relatively large proportion (14.3%) who had had symptoms for over 10 days before presenting to the emergency department and being admitted.

**Table 2:** Days from symptoms onset to hospital admission, including in nosocomial cases.

**Days**	***N***	**%**	**Cumulative %**
Nosocomial	28	1.1	1.1
0	258	10.5	11.7
1	375	15.3	27.0
2	262	10.7	37.7
3	172	7.0	44.7
4	170	6.9	51.7
5	164	6.7	58.4
6	158	6.5	64.8
7	235	9.6	74.4
8	162	6.6	81.0
9	114	4.7	85.7
≥10	350	14.3	100
Total	2,448	100.0	

Regarding the median days from symptoms onset to diagnosis, and not counting patients with nosocomial infections, this interval was 4 (SD 4.6) days. Patients acquiring COVID-19 by nosocomial transmission had been admitted for a median 8.5 (7.2) days, with no differences between the 21 men and 7 women in this group.

Among the 364 patients admitted to the ICU (Table 3), there was a preponderance of men (68%). Mean age was 63.7 (SD 13) years, with no difference according to gender (student's *t*-test *p* > 0.05). About 20% of the ICU patients presented coinfections (in addition to COVID-19), being almost 6% higher than co-infections found in other hospitalized patients. Multidrug-resistant germs were behind approximately 13% of the infections, and *C. auris* was responsible for 4%. The percentage of patients with colonization was higher, reaching 30.8%, while the mortality rate was 31.04%. The median interval between symptoms onset and ICU admission was 8.4 (SD 7.7) days, and it is worth noting the fact that 60% of the patients admitted to the ICU did not exceed a stay of 10 days in the ICU.

**Table 3:** Characteristics of patients admitted to the ICU for COVID-19, February 2020 to February 2021.

		***N***	**%**	**Cumulative %**
**Gender**	Men	247	67.9	67.9
	Women	117	32.1	100.0
**Coinfection**	No coinfection	289	79.4	79.4
	*Candida auris* alone	14	3.8	83.2
	MDR germs alone	46	12.6	95.9
	Both	15	4.1	100.0
**Colonization**	No colonization	252	69.2	73.9
	*Candida auris* alone	29	8.0	80.5
	MDR germs alone	59	16.2	93.1
	Both	24	6.6	100.0
**Charlson comorbidity**	0	95	26.1	26.1
	1	104	28.6	54.7
	2	80	22.0	76.7
	3	32	8.8	85.5
	4	23	6.3	91.8
	5	5	1.4	93.1
	6	15	4.1	97.3
	≥7	10	2.7	100.0
**Length of ICU stay, days**	0–10	211	58.0	58.0
	11–20	73	20.1	78.1
	21–30	21	5.8	83.9
	31–40	19	5.2	89.1
	41–50	16	4.4	93.5
	51–60	3	0.8	94.3
	61–70	2	0.5	94.8
	71–80	1	0.3	95.1
	>81	18	4.9	100.0
**Type of discharge**	No exitus	251	69.0	69.0
	Exitus	113	31.0	100.0

*MDR, multidrug-resistant; Both, MDR and Candida auris*.

Table 4 presents the results of the analysis of variables associated with mortality among COVID-19 patients admitted to the ICU. The strongest association observed was for Charlson comorbidity, especially for scores of 7 or more (OR 8.14 95% CI 2.25, 29.42), followed by Charlson scores of 6 or more (OR 6.05, 95% CI 1.56, 23.48), infection with multidrug-resistant germs (OR 3.52, 95% CI 1.64, 7.58), and coinfection with both multidrug-resistant germs and *C. auris* (OR 3.07, 95% CI 1.11, 8.52).

**Table 4:** Multivariable logistic regression for mortality in patients admitted to the ICU with COVID.

	**β**	***SE***	**Wald**	***df***	***P*** **-value**	**OR**	**95% CI**	
Days from symptoms onset to admission	−0.04	0.03	2.39	1	0.12	0.96	0.91	1.01
Male sex	−0.12	0.31	0.15	1	0.70	0.89	0.48	1.64
Age (years)	0.001	0.01	0.01	1	0.92	1.00	0.98	1.03
Charlson (points)			14.65	7	0.04			
Charlson (1)	0.98	0.56	3.08	1	0.079	2.67	0.89	7.98
Charlson (2)	1.49	0.58	6.47	1	0.011	4.42	1.41	13.88
Charlson (3)	0.63	0.74	0.73	1	0.39	1.88	0.44	8.02
Charlson (4)	1.41	0.74	3.68	1	0.055	4.09	0.97	17.26
Charlson (5)	1.75	0.90	3.80	1	0.051	5.73	0.99	33.08
Charlson (6)	1.80	0.69	6.78	1	0.009	6.06	1.56	23.48
Charlson (≥7)	2.10	0.66	10.24	1	0.001	8.14	2.25	29.42
Infection			13.48	3	0.004			
*C. auris* alone	1.16	0.62	3.51	1	0.061	3.18	0.95	10.65
MDR germs alone	1.26	0.39	10.35	1	0.001	3.52	1.64	7.58
*C. auris* + MDR germs	1.12	0.52	4.65	1	0.031	3.07	1.11	8.52
Colonization			2.04	3	0.57			
*C. auris* alone	0.76	0.56	1.83	1	0.18	2.13	0.71	6.37
MDR germs alone	0.30	0.40	0.56	1	0.45	1.35	0.61	2.99
*C. auris* + MDR germs	0.13	0.58	0.05	1	0.82	1.14	0.37	3.56
Length of ICU stay (days)	0.01	0.02	0.84	1	0.36	1.01	0.98	1.05
Constant	−3.35	0.96	12.12	1	<0.001	0.04		

*CI, confidence interval; df, degrees of freedom; MDR, multidrug-resistant; OR, odds ratio; SE, standard error*.

Regarding the factors associated with a higher risk of death in the total sample (Table 5), the most important was coinfection, especially with *C. auris* (OR 4.64, 95% CI 3.12, 6.91) but also with multidrug-resistant germs. And interestingly, here the colonisations by *Candida auris* were significant (OR: 2.36 95% CI 1.58. 3.52), as well as by MR germs and by the association of both (OR: 1.65 95% CI 1.65, 2.35). Charlson indexes of 6 points and 7 or more points were also linked to higher mortality. Notably, age had virtually no statistically significant influence when the effect of comorbidity, gender, co-infections, and average hospital stay were removed.

**Table 5:** Multivariable logistic regression for mortality in total sample of patients admitted to hospital for COVID-19.

	**β**	***SE***	**Wald**	***df***	***P***-**value**	***OR***	**95% CI**	
Days from symptoms onset to admission	0.003	0.011	0.091	1	0.76	1.00	0.98	1.02
Male sex	0.009	0.11	0.006	1	0.94	1.01	0.82	1.25
Age (years)	−0.006	0.003	4.11	1	0.04	0.99	0.99	1
Charlson (points)			15.86	7	0.026			
Charlson (1)	0.26	0.15	3.10	1	0.078	1.29	0.97	1.72
Charlson (2)	0.32	0.16	3.90	1	0.048	1.38	1.00	1.91
Charlson (3)	0.18	0.20	0.81	1	0.37	1.20	0.81	1.79
Charlson (4)	0.039	0.20	0.039	1	0.84	1.04	0.71	1.52
Charlson (5)	−0.081	0.42	0.038	1	0.85	0.92	0.41	2.09
Charlson (6)	0.88	0.29	9.17	1	0.002	2.42	1.37	4.28
Charlson (≥7)	0.81	0.36	5.11	1	0.024	2.25	1.11	4.53
Infection			70.29	3	0			
*C. auris* alone	1.54	0.20	57.33	1	0	4.64	3.12	6.91
MDR germs alone	0.75	0.16	22.27	1	0	2.11	1.55	2.88
*C. auris* + MDR germs	0.086	0.25	0.12	1	0.73	1.09	0.66	1.79
Colonization			25.50	3	0			
*C. auris* alone	0.86	0.20	17.70	1	0	2.36	1.58	3.52
MDR germs alone	0.40	0.15	6.83	1	0.009	1.49	1.10	2.00
*C. auris* + MDR germs	0.50	0.18	7.84	1	0.005	1.65	1.16	2.35
Length of hospital stay (days)	0.01	0.027	0.105	1	0.74	1.01	0.95	1.06
Constant	−2.05	0.25	67.96	1	0	0.13		

*CI, confidence interval; df, degrees of freedom; MDR, multidrug-resistant; OR, odds ratio; SE, standard error*.

## Discussion

To our knowledge, this is one of the few prospective studies with up to 1 year of follow-up assessing the influence of multidrug-resistant pathogens on mortality in patients hospitalized for COVID-19. The pandemic has interrupted life in general and hospital routines in particular, with many centers compelled to provide diagnostic and treatment services exclusively to those with suspected or confirmed COVID-19. This narrow focus has been applied without sufficient consideration for other possible factors, including colonization and infection. There are many published studies and even meta-analysis on predictors of mortality (13), but the vast majority are small in size and practically none include colonizations of germs that are so important today.

The crude mortality rate in our study population of inpatients during the first year of the pandemic was 19.4%, within the range of other studies reporting rates of 4–28%, although generally other studies have had smaller populations (14), especially with regard to those admitted to the ICU (15, 16). In our study, the median interval from onset to admission, not counting those with nosocomial infections, was slightly shorter at 4.6 days than the 7-day interval reported in a retrospective multicenter cohort study from the Netherlands (17), which may reflect a faster and more severe evolution in our patients. Unlike other studies (18), we did not find a higher risk in men after adjusting for comorbidity and age.

Compared to the literature, the mean length of hospital stay was relatively high at 11.6 days. A US study based on administrative data observed a mean stay of just 7 days (in a selected population of adults) (19), similar to another retrospective study using an administrative database in Australia (20), and another in a small sample of patients precisely in Wuhan but in only 1 month of 2020 (21).

According to the WHO, there is currently an alarmingly rapid propagation of multidrug-resistant and pan-resistant bacterias (also known as “superbacterias”), causing infections that cannot be treated with existing antimicrobials (22). While antibiotics are not effective for COVID-19, they are nevertheless administered to patients with suspected or confirmed COVID-19 infections, for several reasons. For one, it is difficult to rule out a bacterial coinfection at the time of presentation, and moreover, there is a risk of a secondary bacterial infection during the course of the disease. Because researchers have extrapolated information on the increased mortality in patients with bacterial superinfection during flu pandemics, empirical antibiotics are used for patients with severe COVID-19 (23). In our study, just 21% of our COVID-19 patients had a coinfection, and this was associated with an added risk for the patient. On the other hand, a much larger proportion—63%—had a Charlson index of 1 point or more.

Azithromycin is probably one of the most commonly administered treatments for inpatients with COVID-19, both in general (24–26) and in our center. This antibiotic belongs to the family of macrolides, which is used against a wide range of bacterial diseases; however, it has also been shown to have antiviral and immunomodulating properties that could be of interest in viral infections like COVID-19. Its value for preventing coinfections is unknown.

Co-infections continue to be important in COVID-19 mortality, as has been progressively established (15, 16). However, in this study, mortality was also associated with colonization by both MR and *C. auris* germs or both simultaneously, a situation that has not been reported in published studies so far. It may be that the germ in the carrier state is able to enter the human body when it finds a route of access or a significant decrease in immunity and ends up producing a co-infection as has been described in other infectious conditions (8).

One reflection of the greater severity and difficulty for treating COVID-19 is the fact that the mean length of hospital stay was longer than the overall average (7.2 days) for all inpatients in the year prior to the pandemic. The additional 4.5 days they spent in hospital could increase the risk of infections. Something similar occurred in the patients admitted to the ICU, indicating greater clinical complexity and a subsequently higher risk of coinfections and colonization, an added risk for nosocomial infections.

It is known that the outbreaks of *C. auris* that occur in patients with COVID admitted to the ICU are very important and their containment is very difficult, as is widely documented (27, 28). Due to the nature of hospital care in COVID-19, health professionals often wear the same personal protective equipment to attend different patients. This practice may have increased the risk of horizontal transmission, even though these patients have always been indicated for isolation protocols. However, in our case we cannot document the horizontal transmission due to difficulties cloning *C. auris* and/or multidrug-resistant germ chains.

The consequences of colonization/infection by multidrug-resistant bacteria have not yet been evaluated in patients with COVID-19, but they could be concerning for several reasons. First of all, these patients already experience immune system dysfunctions, both because of the disease itself and the immunomodulating therapies administered to treat it. Secondly, hospital COVID-19 cases have a profile characterized by advanced age, comorbidities, prolonged hospital stays, and numerous invasive procedures, all of which are risk factors for colonization or infection by multidrug-resistant bacteria.

There seems to be evidence that some serological markers of inflammation associated with bacterial infections, like elevated procalcitonin and C-reactive protein, can appear in patients with COVID-19 (29) without any bacterial coinfection. However, in our study we used only the germ cultures and their corresponding resistance, so this limitation does not apply.

Another important variable we assessed was comorbidity, adjusted for age, sex, and infection/colonization. Comorbidity has been studied in an *ad hoc* way conjunction with age in patients with COVID-19 (13, 30–32), but in this patient group, it is more appropriate to use standardized comorbidity indicators that are quantitatively associated with mortality, such as the Charlson comorbidity index. One study of COVID-19 did use that indicator; however, the sample was limited to people aged 40–85 years (33), unlike ours; moreover, when age was adjusted for comorbidity, it did not influence mortality.

Many of the components of the Charlson index have been individually linked to higher mortality in COVID-19, including diabetes (13, 34) and chronic obstructive pulmonary disease (35), among others, as cardiovascular diseases (36). Thus, it is unsurprising that this tool is useful for measuring composite mortality risk in this population, even after adjusting for other factors.

Among the main limitations of this study, we included patients with nosocomial infections. However, we considered that these cases generally originate in routine hospital activities, and these are often the patients with the poorest outcomes. Our sample was also quite heterogeneous, and it was sometimes difficult to obtain data for patients were referred from private hospitals. It is even possible that these referrals led to an increase in coinfections and colonization in some patients who were carrying these infections upon admission to our center.

In most cases, patients were not assessed for colonization on the same day of admission, so it is not possible to conclude whether all cases of colonization were acquired in hospital. Moreover, detecting coinfections and colonization often depends on clinical suspicion or investigation, as the symptoms may be masked by those caused by COVID-19 itself. For the same reason, patients did not always distinguish the onset of symptoms, confusing them with the symptoms of respiratory pathologies or others. When they presented to the emergency department, they often did not suspect that they had COVID-19 and had delayed seeking care until their condition had deteriorated substantially.

The implications of our results include the need to perform more controls in patients with COVID-19 monitoring not only infections, but also colonizations, all aimed at optimizing isolation measures. In that respect, control of MR germs (including *Candida auris*) is essential. Moreover, there is a clear need to characterize the difference in outcome with colonization vs. infection with MDR.

Obviously, it is also necessary to continue assessing the precise impact of COVID-19 in relation to the administration of antimicrobials, infection control, and development of appropriate public health strategies to prevent the propagation of antimicrobial-resistant pathogens.

All in all, much remains to be studied, but the practical importance that can be generated for the benefit of the patient makes it worth the effort.

## Data Availability Statement

The raw data supporting the conclusions of this article will be made available by the authors, without undue reservation.

## Ethics Statement

The studies involving human participants were reviewed and approved by Ethics and Research Committee of University General Hospital of Valencia. Written informed consent for participation was not required for this study in accordance with the national legislation and the institutional requirements.

## Author Contributions

All authors listed have made a substantial, direct and intellectual contribution to the work, and approved it for publication.

## Conflict of Interest

The authors declare that the research was conducted in the absence of any commercial or financial relationships that could be construed as a potential conflict of interest.

## Publisher's Note

All claims expressed in this article are solely those of the authors and do not necessarily represent those of their affiliated organizations, or those of the publisher, the editors and the reviewers. Any product that may be evaluated in this article, or claim that may be made by its manufacturer, is not guaranteed or endorsed by the publisher.
